# Study on Adsorption of Cd in Solution and Soil by Modified Biochar–Calcium Alginate Hydrogel

**DOI:** 10.3390/gels10060388

**Published:** 2024-06-06

**Authors:** Shuyue Wang, Yajun Wang, Xinyi Wang, Sijia Sun, Yanru Zhang, Weixiong Jiao, Dasong Lin

**Affiliations:** Agro-Environmental Protection Institute, Ministry of Agriculture and Rural Affairs, Tianjin 300191, China; vivraxxvvy_w@163.com (S.W.);

**Keywords:** modified biochar, calcium alginate, Cd pollution, in situ passivation

## Abstract

Contamination with cadmium (Cd) is a prominent issue in agricultural non-point source pollution in China. With the deposition and activation of numerous Cd metal elements in farmland, the problem of excessive pollution of agricultural produce can no longer be disregarded. Considering the issue of Cd pollution in farmland, this study proposes the utilization of cross-linked modified biochar (prepared from pine wood) and calcium alginate hydrogels to fabricate a composite material which is called MB-CA for short. The aim is to investigate the adsorption and passivation mechanism of soil Cd by this innovative composite. The MB-CA exhibits a higher heavy metal adsorption capacity compared to traditional biochar and hydrogel due to its increased oxygen-containing functional groups and heavy metal adsorption sites. In the Cd solution adsorption experiment, the highest Cd^2+^ removal rate reached 85.48%. In addition, it was found that the material also has an excellent pH improvement effect. Through the adsorption kinetics experiment and the soil culture experiments, it was determined that MB-CA adheres to the quasi-second-order kinetic model and is capable of adsorbing 35.94% of Cd^2+^ in soil. This study validates the efficacy of MB-CA in the adsorption and passivation of Cd in soil, offering a novel approach for managing Cd-contaminated cultivated land.

## 1. Introduction

Soil is the material foundation for human survival, and good soil quality is significant for maintaining the basic functions of ecosystems. Heavy metal pollution is one of the biggest environmental pollution problems worldwide [1]. Unlike other pollutants, heavy metals are prone to accumulation in organisms and do not have biodegradability, ultimately leading to disease or death [2]. Cd is a harmful element to the human body and a non-essential element for plants [3,4]. It is easy to transfer and has strong toxicity. In addition, Cd pollution is also highly harmful and the most widespread metal pollution in China [5,6]. The Cd content in Chinese paddy soil ranges from 0.01 to 5.50 mg·kg^−1^, with a median of 0.23 mg·kg^−1^. Compared with the other provinces, the Cd content of the paddy soil in Hunan (0.73 mg·kg^−1^), Guangxi (0.70 mg·kg^−1^), and Sichuan (0.46 mg·kg^−1^) provinces is higher. Mining, smelting, sewage farming, air pollution, and the application of Cd-containing fertilizers are the main causes of Cd pollution in many paddy fields and dry lands in China [7]. Cd pollution in soil has toxic effects on soil organisms, affecting soil microbial populations, community structure, biochemical reactions, and soil enzyme activity [8,9]. For plants, Cd interferes with the absorption of nutrients, inhibiting photosynthesis, causing oxidative stress and gene damage, and affecting plant growth metabolism [10,11]. Normally, if the Cd content in the soil exceeds 8 mg·kg^−1^, most crops will exhibit visible Cd toxicity symptoms.

Applying in situ passivation materials in cultivated land is an effective way to reduce the bioavailability of Cd in the soil [12,13]. Traditional passivated materials include biochar, limestone, shell powder, silicate, zeolite, phosphate rock powder, etc. [14,15,16,17,18]. The main principle of their remediation of Cd-contaminated soil is to adjust the soil pH or combine with Cd ions to form stable compounds. However, the conventional passivation materials often cause issues such as soil compaction and secondary pollution, while the utilization of unmodified biochar is also constrained by its functionality, leading to unsatisfactory performance in heavy metal removal. In addition, the remediation effect of traditional materials on Cd ions is not stable due to the complexity of soil and water systems in nature [19].

Modified biochar has emerged as a prominent research focus in the field of environmental adsorption materials in recent years, owing to its exceptional adsorption capacity [20,21,22]. It is prepared by modifying traditional biochar through ball milling, chemical reactions, and other modification methods. Compared with traditional biochar, modified biochar has a larger specific surface area and more abundant oxygen-containing functional groups, providing many heavy metal adsorption sites [23]. Li and Shi found that the specific surface area, pore volume, and microporous volume of iron and dicyandiamide co-modified walnut shell biochar were 967.1084 m^2^·g^−1^, 0.7425 cm^3^·g^−1^, and 0.4624 cm^3^·g^−1^, respectively, which were 3.39, 6.42, and 8.81 times that of the original biochar [24]. Wang et al. found that carboxymethyl cellulose combined with nano zero-valent zinc modification can make the surface of biochar (nZVZ-CMC-PMBC) rougher, with a larger specific surface area and more developed pore size. Simultaneously, zero-valent zinc enters spherical particles and forms a nano-metal thin plate structure which strengthens the adsorption effect of the composite material on pollutants [25]. Jin et al. found that the adsorption capacity of modified biochar for heavy metal arsenic was significantly increased after modifying with potassium hydroxide [26]. Han et al. found that the surface adsorption sites of biochar increased and the adsorption capacity for Cr^6+^ was significantly improved after modifying with FeCl_3_ [27]. Liang et al. found that the maximum adsorption capacity of heavy metals by biochar modified with amorphous MnO_2_ was higher than that of unmodified biochar [28]. Liang et al. added thiol-modified sepiolite to Cd contaminated farmland soil, resulting in a 65.4% to 77.9% decrease in Cd content in rice [29]. In actual soil remediation, modified biochar is easily able to cause heavy metal migration and desorption due to its small particle size and aging, which is worthy of further study.

In recent years, hydrogel materials [30,31,32,33,34] have been widely used in the treatment of heavy metal ions due to their wide source of raw materials, low cost, strong adsorption capacity for metal ions, and other characteristics [35]. Alginate saline gel has good hydrophilicity and biocompatibility, and its richness in surface functional groups (such as carboxyl and hydroxyl) can capture metal ions effectively [36]. The unique swelling property of hydrogel makes heavy metal ions adsorb not only on the surface of the hydrogel, but also on the three-dimensional network structure during the removal process [37]. Compared with the traditional methods, hydrogel materials show obvious advantages in the adsorption of heavy metal ions, such as environmental friendliness, microstructure designability, and biodegradability, etc. Chan et al. [38] prepared a DNA–chitosan hydrogel, which can effectively combine with Hg^2+^; the maximum adsorption capacity of Hg^2+^ is 50 mg·g^−1^. Yetimoglu et al. [39] found that the Pb^2+^ and Cd^2+^ adsorbed on AMPSG (guanidine-modified 2-acrylamido-2-methylpropan sulfonic acid)/AAc(acrylic acid)/NVP(N-vinylpyrrolidone)/HEMA(2-Hydroxyethyl methacrylate) hydrogel can be effectively desorbed through acid leaching, and the regenerated AMPSG/AAc/NVP/HEMA hydrogel did not reduce its adsorption properties. The incorporation of organic and inorganic materials, such as carbon-modified tubes, graphene, metal and metal oxides, silica-based materials, etc., into the gel system has garnered significant attention from researchers due to its potential for enhancing hydrogel performance in terms of swelling behavior, mechanical properties, and adsorption capacity [40,41]. Li et al. [42] designed a sodium lignosulfonate–guar gum composite hydrogel, which had excellent adsorption performance for heavy metals in soil. The maximum adsorption capacity of Cu^2+^ and Co^2+^ are 709.0 mg·g^−1^ and 601.00 mg·g^−1^, respectively. Wong et al. [43] developed a nano hydroxyapatite–cellulose hydrogel composite material, which removed 70.24%, 57.74%, 48.56%, 27.33%, and 25.98% of Cu^2+^, Pb^2+^, Fe^2+^, Cd^2+^ and Zn^2+^ ions from palm oil factory wastewater, respectively. Yin et al. [44] prepared a modified xanthan gum–hydroxyapatite composite hydrogel (XG-g-PAA/HAP), and more than 90% of metal ions were removed within 30 min. Zhang et al. [45] mixed a dissolved cellulose solution, a TEMPO (2,2,6,6-tetramethylpiperidinyl-1-oxide)-oxidized cellulose nanofiber (TOCN) dispersion, and an alkali lignin solution in a NaOH–urea aqueous solution to prepare a composite hydrogel based on lignocellulose. The maximum adsorption amount of Cu^2+^ on the composite hydrogel reached 541 mg·g^−1^. In addition, the presence of TOCN and lignin made the composite hydrogel show high strength performance.

**Figure 1:**
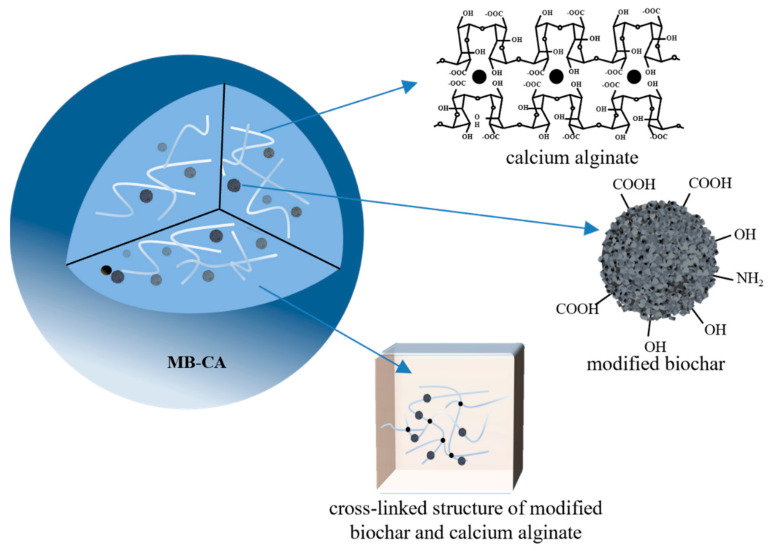
The fundamental structure of MB-CA.

The main waste of furniture production is pine sawdust, which often has a higher lignin content and lower ash content compared to agricultural straw. Therefore, it is an ideal material for preparing modified biochar. In this research, the authors aim to cross-link modified biochar and calcium alginate hydrogel to prepare a modified biochar–calcium alginate hydrogel composite, referred to as MB-CA, whose basic structure is shown in Figure 1. This composite aims to address Cd pollution in farmland by exploring its adsorption and passivation mechanism for soil Cd. Wang et al. [36] successfully synthesized a novel composite material with significant advantages by impregnating ball-milled biochar with calcium alginate particles through an innovative approach. This material not only exhibits enhanced water and fertilizer retention capacity, which can effectively lock up water and nutrients in the soil and reduce their loss, but also has good controlled-release properties, releasing nutrients on demand and providing stable and long-lasting nutrient support for plant growth. Modification of biochar is often required to improve some of its properties. Pretreatment of biomass with phosphoric acid (H_3_PO_4_) for biochar production can improve carbon (C) retention, porosity structure, and the sorption ability of biochar [46]. In this paper, the plan is to validate the feasibility of MB-CA remediation of Cd-contaminated soil through verification of adsorption kinetics, soil cultivation, and other experimental methodologies.

## 2. Results and Discussion

### 2.1. Surface Morphology and Functional Groups of MB-CA

**Figure 2:**
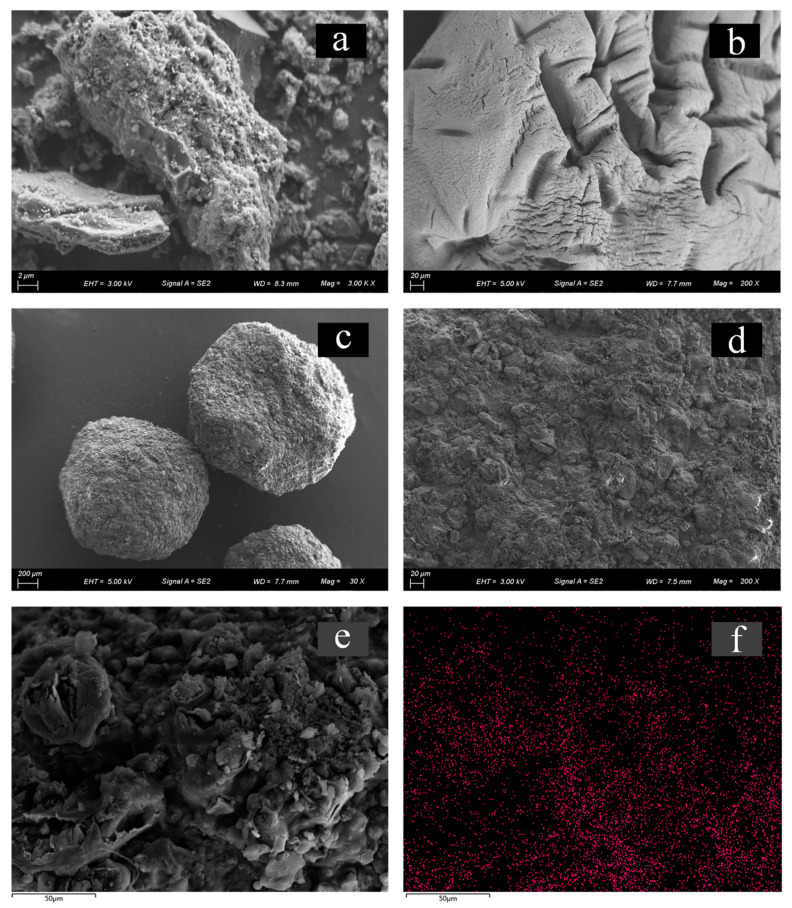
Surface morphology of modified biochar and MB-CA. (**a**) Modified biochar with magnification of 3 k (2 μm of scale bar); (**b**) calcium alginate hydrogel with magnification of 200 (20 μm of scale bar); (**c**) MB-CA with magnification of 30 (200 μm of scale bar); (**d**) MB-CA with magnification of 200 (20 μm of scale bar); (**e**) MB-CA after Cd^2+^ adsorption (50 μm of scale bar); and (**f**) the distribution of Cd^2+^ corresponding to Figure 2e.

Figure 2a,b show the apparent morphology of modified biochar at different sizes, and the rough surface formed by pyrolysis and the modification of biomass can be clearly seen. The surface microstructure of MB-CA is shown in Figure 2c,d. It can be found that the surface of the composite has deep and wide wrinkles and grooves after lyophilization. In addition, the BET surface area and average pore diameter were both measured; their values were 78.43 m^2^·g^−1^ and 4.67 nm, respectively. The results suggest that the adsorption of Cd^2+^ by the composite is primarily attributable to complexation reactions between Cd^2+^ and the functional groups, rather than being predominantly driven by physical adsorption. To further investigate the functional groups present in the composite, FT-IR analysis was conducted. Figure 2e,f show the distribution of Cd^2+^ on the surface of the composite material after the adsorption of Cd^2+^.

**Figure 3:**
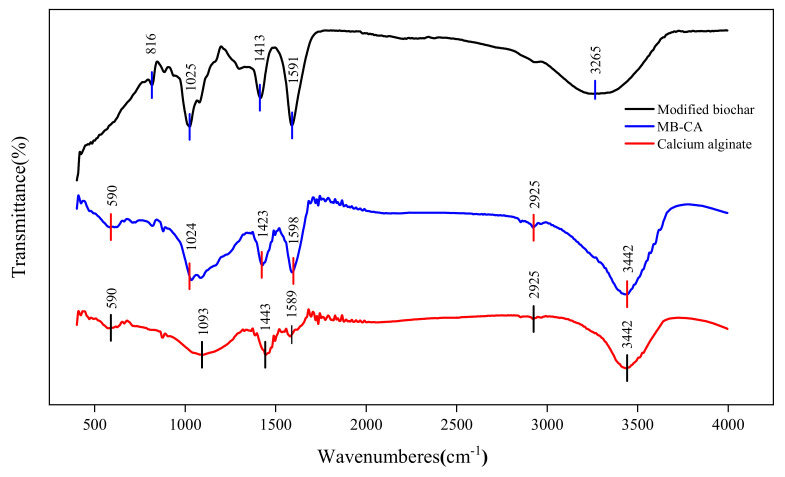
FTIR results of modified biochar, MB-CA, and calcium alginate.

The functional groups of MB-CA, calcium alginate, and the modified biochar were characterized by FT-IR spectra. As shown in Figure 3, the addition of modified biochar provides more types and quantities of functional groups in the composite. The broad peak at 3442 cm^−1^ before adsorption belongs to the free -OH (hydroxyl) in the molecule, indicating the presence of a large amount of -OH (hydroxyl) on the surface of the composite material. The peak at 2925 cm^−1^ is the vibration of aromatic C-H (hydrocarbon bond); the absorption peaks at 1589 cm^−1^ and 1436 cm^−1^ are generated by the stretching vibration of C=O (carbon oxygen double bond) and C-O (carbon oxygen single bond) in -COOH (carboxyl group), respectively, indicating the presence of a large amount of -COOH groups (carboxyl groups) on the surface of the composite material, which can provide a large number of adsorption sites and facilitate the adsorption of more Cd^2+^ by the composite material; the stretching at 1024 cm^−1^ is caused by the C-O (carbon oxygen single bond) functional group. Complexation is especially significant in wood charcoal or straw charcoal with a low inorganic mineral content. Teng et al. [47] characterized pine charcoal by FTIR, and found that there were characteristic bands such as -OH, -C-H, -C-O, -C=C, -COOH, and phenolic -OH, etc. Surface functional group complexation is the main mechanism of Cd adsorption, and the typical complexation reaction formula is: C-OH+Cd^2+^+H_2_O→C-OCd^+^+H_3_O^+^, 2C-COOH+Cd^2+^→(C-COO)_2_Cd+2H^+^ [48], etc.

### 2.2. Thermogravimetric Analysis of MB-CA

**Figure 4:**
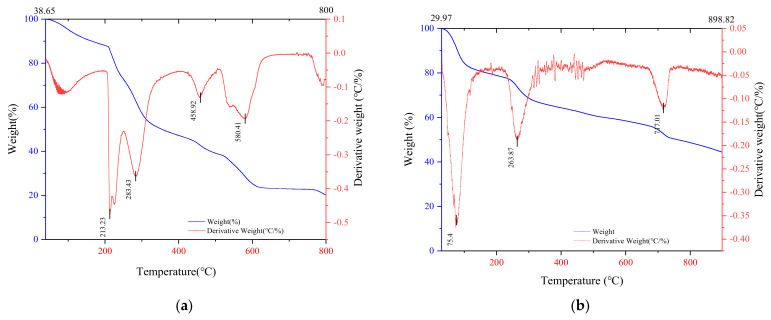
Weightlessness curve of calcium alginate gel and MB-CA. (**a**) Calcium alginate gel; (**b**) MB-CA.

The thermal stability of the calcium alginate gel and MB-CA was verified through a thermogravimetric experiment. As shown in Figure 4b, the weightlessness curve of MB-CA exhibited three distinct weight loss phenomena at temperatures of 75.4 °C, 263.87 °C, and 717.01 °C, respectively. It is believed that these peaks correspond to the thermal degradation of water, calcium alginate, and activated carbon. At the end of the test, more than 40% of the solid residue was still left, which was considered to be mainly modified biochar residue.

### 2.3. The Effect of pH on the Adsorption of Cd^2+^ by MB-CA

Five 50 mL portions of Cd(NO_3_)_2_ solution at a concentration of 50 mg/L were prepared and the pH of each solution was adjusted to 2, 3, 4, 5 and 6, respectively. Subsequently, 0.06 g of MB-CA was added to each solution. The solutions were then magnetically agitated at room temperature (25 ± 1) °C for a duration of 24 h. Afterward, the concentrations of Cd^2+^ in the solutions as well as their respective pH values were measured. A total of 3 sets of parallel experiments were conducted and the results were averaged.

Table 1 shows the effect of pH on the adsorption of Cd^2+^. The adsorption capacity of MB-CA on Cd^2+^ was significantly enhanced as the pH increased from 2 to 5, leading to an increase in the concentration of Cd^2+^ within the material from 3.65 ± 1.67 mg·g^−1^ to 48.52 mg·g^−1^. The adsorption capacity exhibited a slight decrease at pH 6. When the pH is 2, a significant abundance of positively charged H^+^ ions surround the surface of MB-CA in the solution, while Cd exists as cations in the aqueous solution and competes with H^+^ for adsorption sites, leading to a reduction in Cd^2+^ adsorption capacity. As the pH increases, the concentration of H^+^ in the solution decreases, leading to an enhancement of MB-CA adsorption capacity which reaches its peak at pH 5 and the highest Cd^2+^ removal rate can reach 85.48%. Analyzing the reason for the above phenomenon, it may be that under strong acidic conditions, a large amount of H^+^ in the solution will occupy limited binding sites and compete with Cd^2+^ for adsorption, reducing the Cd^2+^ removal rate [49]; with the increase in pH, the amount of H^+^ decreases, which exposes a large number of binding sites on the surface of the material, and the adsorption capacity is also increased [50]. Interestingly, this experiment also revealed that the composite material effectively raised the pH of the solution beyond 6 when immersed in a solution with a pH ranging from 3 to 6; this observation suggests that the composite material possesses an alkalizing effect on solutions, thereby offering a novel perspective on Cd passivation mechanisms in soil. It is necessary to verify the passivation effect of the MB-CA on Cd and the pH-raising effects in the soil.

### 2.4. Dynamic Adsorption of MB-CA

**Figure 5:**
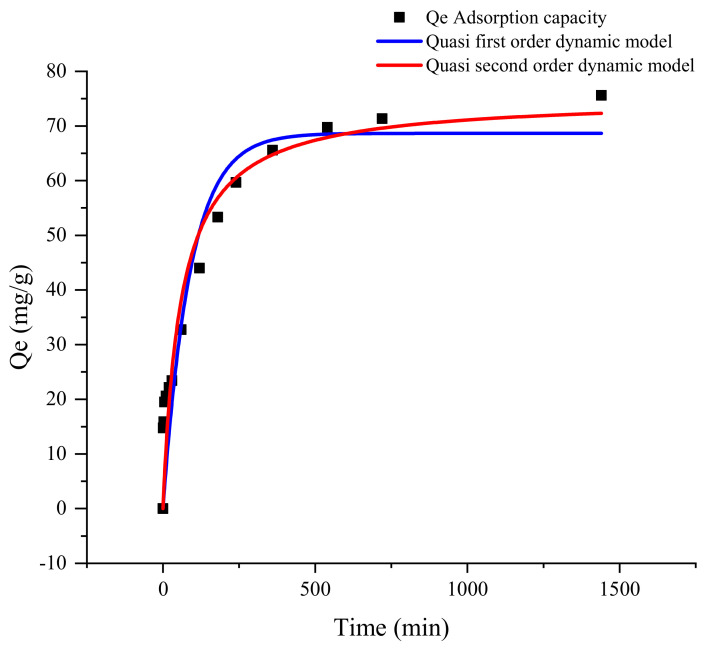
Kinetic adsorption diagram of MB-CA.

The data presented in Figure 5 demonstrate that the composite material exhibits a substantial initial adsorption capacity for Cd^2+^, followed by a gradual attainment of adsorption equilibrium. This experiment studied the kinetic behavior of MB-CA adsorption of Cd^2+^ by fitting dynamic adsorption data. Quasi-first-order kinetic models and quasi-second-order kinetic models were applied to fit the data, and the equations are shown in Equations (1) and (2)

lg(Q_e_ − Q_t_) = lgQ_e_ − k_1_/2.303t (1)

t/Q_t_ = 1/k_2_q^2^e + 1/Q_e_ (2)

In the formula, Q_t_ (mg·g^−1^) and Q_e_ (mg·g^−1^) are the adsorption capacities at time t (min) and equilibrium time of the adsorbent, respectively. k_1_ (min^−1^) and k_2_ [g/(mg·min)] are the rate constants of the quasi-first-order and quasi-second-order kinetic equations, and t (min) is the adsorption time [51]. The kinetic experimental data of MB-CA adsorption is shown in Figure 5.

The initial adsorption rate of Cd^2+^ by MB-CA is rapid, primarily attributed to the outer layer of the gel absorbing Cd^2+^, followed by a deceleration in the adsorption rate. At this stage, Cd^2+^ is predominantly adsorbed by the modified biochar and calcium alginate inside the composite. The dynamic fitting results are presented in Table 2.

According to Table 2, the correlation coefficient of the quasi-second-order kinetic model for MB-CA is 0.915, which is higher than the correlation coefficient of the quasi-first-order dynamic model (0.868). Additionally, the equilibrium adsorption amount fitted by the quasi-second-order kinetic model closely approximates the actual value. According to the liquid–solid adsorption theory, diffusion is a rate control step.

### 2.5. The Results of Soil Culture Experiment

**Figure 6:**
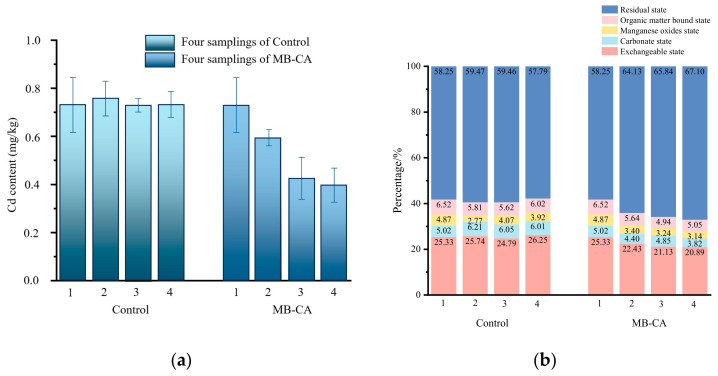
The influence of MB-CA on the concentration and speciation of Cd in soil. (**a**) The Cd^2+^ content; (**b**) chemical species of Cd.

The results of our 30-day soil culture experiment indicate that the application of the composite material led to a gradual increase in soil pH, resulting in a rise from 6.7 to 7.1. Compared to the blank control group (MB-CA not applied), as shown in Figure 6, the application of MB-CA resulted in a reduction in Cd^2+^ concentration in contaminated soil from 0.73 mg·kg^−1^ to 0.40 mg·kg^−1^ after conducting four samplings. Additionally, the composite material also influenced the distribution of Cd in the soil, with an increase in the residual state from 58% to 67%, and a decrease in the exchangeable state from 25% to 21%. These results indicate that MB-CA effectively mitigates the total content and bioavailability of Cd in soil. This indicates that MB-CA can effectively reduce the toxicity and bioavailability of Cd in soil.

## 3. Conclusions

MB-CA exhibits a higher abundance of oxygen-containing functional groups and heavy metal adsorption sites compared to calcium alginate. A pH ranging from 3 to 6 exhibits a favorable adsorption effect. The adsorption of Cd^2+^ by the composite is more consistent with the quasi-second-order kinetic model. The Cd^2+^ concentration and bioavailability of soil were significantly reduced by the application of MB-CA, demonstrating its significant potential for reducing the risk of agricultural products that exceed regulatory standards. This study proposes the cross-linking preparation of modified biochar with calcium alginate to form MB-CA composites. This composite material combines the adsorption properties of biochar with the gel properties of calcium alginate, which makes it show unique advantages in the field of heavy metal pollution remediation. This innovative material combination provides new possibilities for soil heavy metal pollution remediation. MB-CA composites were applied in the remediation and treatment of soil Cd pollution. Through multi-level experiments, the adsorption and passivation mechanisms of MB-CA composites on soil Cd were investigated in depth, revealing their practical effects in soil pollution remediation. This application innovation not only expands the application scope of biochar and hydrogel composites, but also provides a new technical means for soil heavy metal pollution remediation.

Future research on heavy metal adsorption by hydrogels can focus on the following aspects: (1) improving the cross-linking technology and preparation process to reduce the cost of hydrogel preparation; (2) selecting and developing new cross-linking agents to enhance the mechanical strength and adsorption capacity of hydrogels; (3) strengthening the research and development of hydrogels with selective adsorption, specific sensitivity to the adsorption environment, and high sensitivity to heavy metal ions, as well as other specific functions of hydrogels; (4) developing detoxification materials, which can make the adsorbed materials with heavy metal ions precipitated from the soil. Overall, the MB-CA composites have excellent adsorption effects and broad application prospects, which are worthy of more in-depth research.

## 4. Materials and Methods

### 4.1. Preparation of Modified Biochar/Modified Biochar Calcium Alginate Hydrogel Composite

Pulverized pine sawdust, with uniform particle size (passing through a 60-mesh sieve), was impregnated with a solution of phosphoric acid. Subsequently, it was dried and loaded into a pyrolysis furnace for slow pyrolysis under a nitrogen atmosphere at final temperatures of 450 °C, 500 °C, 550 °C, or 600 °C. After cooling down, the modified biochar was prepared and stored for later use. The modified biochar was incorporated into a 2% calcium alginate solution and subjected to ultrasound stirring for 60 min, resulting in the formation of a mixed solution containing modified biochar and calcium alginate. The above mixed solution was slowly added into a 0.2 mol·L^−1^ CaCl_2_ solution while stirring, and the mixture was stirred for 60 min until spherical hydrogels precipitated at the bottom of the beaker to obtain the composite material MB-CA. The composite material was subsequently washed with hydrochloric acid and clean water, followed by cooling and drying. The optimal process parameters were selected based on the single indicator of Cd^2+^ adsorption strength per unit mass in a solution of Cd (NO_3_)_2_.

### 4.2. Characterization of Physical and Chemical Properties of Composite Materials MB-CA

By utilizing advanced techniques such as SEM-EDS (scanning electron microscopy–energy spectrum analysis, ZEISS Gemini Sigma 300, Oberkochen, Germany, incident electron beam: 3 and 5 kV), FT-IR (infrared spectroscopy analysis, Thermo Scientific Nicolet iS5 FT-IR, Spectral range: 400–4000 cm^−1^, Waltham, MA, USA), TG-DTG (thermogravimetric differential thermal analysis, Swiss Mettler Toledo TGA/DSC 1/1600, Greifensee, Switzerland, temperature range: 25–800 °C, heating rate: 10 °C/min, atmosphere: nitrogen), ICP-MS (inductively coupled plasma–mass spectrometry iCAP Q, Waltham, MA, USA), etc., the physical and chemical properties of composite materials were comprehensively analyzed. This included examining their apparent morphology, functional groups, pore size distribution, and thermal stability.

### 4.3. Test on the Adsorption Characteristics of Composite Material MB-CA for Cd in Solution

Optimal adsorption conditions for Cd^2+^ in aqueous solutions were determined through batch intermittent adsorption experiments, employing a HCl (1 mol·L^−1^ and 0.1 mol·L^−1^) and NaOH solution (1 mol·L^−1^ and 0.1 mol·L^−1^), respectively, to adjust the initial pH values of various aqueous solutions within the range of 2–6. Subsequently, 0.6 g of freeze-dried composite material was added to a 30 mL solution of Cd(NO_3_)_2_ with a concentration ranging from 20 to 500 mg·L^−1^, and the beaker was placed in a constant temperature shaker at 20–40 °C for continuous agitation. When the adsorption of the reaction system solution reached equilibrium, a water sample was collected approximately 2 cm below the liquid level. The sample was then filtered and diluted to determine the removal efficiency of Cd^2+^ by the appropriate material.

### 4.4. Adsorption Kinetics

The kinetic adsorption experiment of MB-CA was conducted at room temperature (25 ± 1) °C. Under the conditions of initial Cd^2+^ concentration of 50 mg·L^−1^, adsorption time of 1440 min, and system pH of 5 (pH adjusted with sodium hydroxide and hydrochloric acid), 500 mL of Cd(NO_3_)_2_ solution was added to a 1 L beaker. Then, 0.6 g of MB-CA was added to the 500 mL solution of Cd(NO_3_)_2_ and vigorously stirred using magnetic force. At specific time intervals (ranging from 0 min to 1440 min), a sample of 5 mL Cd(NO_3_)_2_ solution was taken and diluted with a 0.24 mol·L^−1^ HNO_3_ solution. Subsequently, the concentration of Cd^2+^ was measured using an atomic absorption analyzer. The samples were collected at 1, 3, 5, 10, 20, 30, 60, 120, 180, 240, 360, 540, 720, and 1440 min (in triplicate), diluted to a concentration of 1 ppm, and then filtered through the membrane for subsequent use. This experiment accurately demonstrates the dynamic adsorption behavior of composite materials and investigates the influence of composite materials on adsorption effectiveness.

### 4.5. Soil Cd Bioavailability in Soil Culture Experiment

Quantitative measurement of Cd-contaminated soil was conducted, and MB-CA (2% of the soil weight) was quantitatively applied. After mixing, the mixture was filled into pots, and soil samples were collected every 7 days. ICP-MS technology was used to detect the content of Cd^2+^ in soil, and the Tessier five-step extraction method was employed to detect the bioavailability of Cd in soil. The adsorption and passivation effects of composite materials on Cd^2+^ in soil cultivation experiments were evaluated using atomic absorption (ThermoFisher ESCALAB 250Xi, USA).

### 4.6. Reagents

The reagents used in this study were shown in Table 3.

## Figures and Tables

**Table 1:** pH changes before and after adsorption of Cd^2+^ by MB-CA.

Initial pH	pH = 2	pH = 3	pH = 4	pH = 5	pH = 6
Final pH	2.32 ± 0.06	6.04 ± 0.15	6.63 ± 0.02	6.71 ± 0.03	6.73 ± 0.01
Qe (mg·g^−1^)	3.65 ± 1.67	43.87 ± 3.15	46.65 ± 0.99	48.52 ± 4.36	44.97 ± 4.45

**Table 2:** Kinetic fitting parameters of MB-CA adsorption of Cd^2+^.

Sample Name	Quasi-First-Order Dynamic Model			Quasi-Second-Order Dynamic Model			Adsorption Capacity at Equilibrium
	Q_e_, _cal_/mg·g^−1^	K_1_ × 10^−3^/min^−1^	R^2^	Q_e_, _cal_/mg·g^−1^	K_1_ × 10^−3^/min^−1^	R^2^	Q_e_/mg·g^−1^
MB-CA	68.657	0.111	0.868	75.254	2.273	0.915	75.583

**Table 3:** Experimental reagents.

Reagent Name	Purity Level	Source
C_6_H_7_NaO_6_ (SA)	Chemical pure	Sinopharm Chemical Reagent Co., Ltd., Shanghai, China
CaCl_2_	Chemical pure	Bodi Chemical Industry Co., Ltd., Tianjin, China
NaOH	Analytial reagent	Sinopharm Chemical Reagent Co., Ltd., Shanghai, China
Cd(NO_3_)_2_·4H_2_O	Analytial reagent	Fuchen Chemical Reagent Co., Ltd., Tianjin, China
NaNO_3_	Analytial reagent	Xinhao Chemical Industry Co., Ltd., Zibo, China
HNO_3_	Analytial reagent	Luxi Chemical Industry Group Co., Ltd., Liaocheng, China
H₃PO₄	Analytial reagent	Taixi Chemical Industry Co., Ltd., Jinan, China
HCl	Analytial reagent	Beiyuan Chemical Industry Group Co., Ltd., Yulin, China

## Data Availability

The data presented in this study are openly available in article.
